# The Odonata of China: a data-driven, open-access resource for biodiversity research and conservation

**DOI:** 10.1093/database/baaf077

**Published:** 2025-12-23

**Authors:** Shao-Yan Pu, Jing-Sheng Lu, Xin-Ling Tao, Zi-Feng Li, Ya-Nan Wang, Hao-Miao Zhang, Xuemei Lu

**Affiliations:** Key Laboratory of Genetic Evolution and Animal Models, Kunming Institute of Zoology, Chinese Academy of Sciences, Kunming, Yunnan 650201, China; Biodiversity Data Center of Kunming Institute of Zoology, Chinese Academy of sciences, Kunming, Yunnan 650201, China; Yunnan Key Laboratory of Biodiversity Information, Kunming Institute of Zoology, The Chinese Academy of Sciences, Kunming 650201, China; Key Laboratory of Genetic Evolution and Animal Models, Kunming Institute of Zoology, Chinese Academy of Sciences, Kunming, Yunnan 650201, China; Biodiversity Data Center of Kunming Institute of Zoology, Chinese Academy of sciences, Kunming, Yunnan 650201, China; Yunnan Key Laboratory of Biodiversity Information, Kunming Institute of Zoology, The Chinese Academy of Sciences, Kunming 650201, China; Key Laboratory of Genetic Evolution and Animal Models, Kunming Institute of Zoology, Chinese Academy of Sciences, Kunming, Yunnan 650201, China; University of Chinese Academy of Sciences, Beijing 100049, China; Key Laboratory of Genetic Evolution and Animal Models, Kunming Institute of Zoology, Chinese Academy of Sciences, Kunming, Yunnan 650201, China; Yunnan Key Laboratory of Biodiversity Information, Kunming Institute of Zoology, The Chinese Academy of Sciences, Kunming 650201, China; University of Chinese Academy of Sciences, Beijing 100049, China; Key Laboratory of Genetic Evolution and Animal Models, Kunming Institute of Zoology, Chinese Academy of Sciences, Kunming, Yunnan 650201, China; Biodiversity Data Center of Kunming Institute of Zoology, Chinese Academy of sciences, Kunming, Yunnan 650201, China; Yunnan Key Laboratory of Biodiversity Information, Kunming Institute of Zoology, The Chinese Academy of Sciences, Kunming 650201, China; Key Laboratory of Genetic Evolution and Animal Models, Kunming Institute of Zoology, Chinese Academy of Sciences, Kunming, Yunnan 650201, China; Yunnan Key Laboratory of Biodiversity Information, Kunming Institute of Zoology, The Chinese Academy of Sciences, Kunming 650201, China; Kunming Zoological Museum of Kunming Institute of Zoology, Chinese Academy of sciences, Kunming, Yunnan 650201, China; Key Laboratory of Genetic Evolution and Animal Models, Kunming Institute of Zoology, Chinese Academy of Sciences, Kunming, Yunnan 650201, China; Biodiversity Data Center of Kunming Institute of Zoology, Chinese Academy of sciences, Kunming, Yunnan 650201, China; Yunnan Key Laboratory of Biodiversity Information, Kunming Institute of Zoology, The Chinese Academy of Sciences, Kunming 650201, China; University of Chinese Academy of Sciences, Beijing 100049, China

## Abstract

Odonata (dragonflies and damselflies) are among the most ancient winged insects, with over 900 species recorded in China, representing the highest global diversity. However, the lack of a centralized database integrating morphological, ecological, and multiomics data has hindered large-scale research and conservation efforts. We present Odonata of China (http://dragonflies.kiz.ac.cn), a comprehensive database compiling taxonomic, biogeographic, phenotypic, and multiomics data for 820 species across 3 suborders, 22 families, and 172 genera. The database features advanced search modules (direct, phylogenetic, and map-based), genomic and transcriptomic data for 20 representative families, and high-resolution images. The platform is constructed based on mainstream open-source technologies, ensuring scalability and reproducibility. Odonata of China provides a critical resource for evolutionary biology, conservation, and ecological studies. By integrating heterogeneous data types and leveraging modern technologies, this database bridges a significant gap in invertebrate biodiversity informatics and supports global initiatives to monitor insect declines.

## Introduction

Odonata is one of the oldest groups of flying insects known to date, encompassing both larvae and adults [1]. The earliest dragonfly fossils date back to the Carboniferous period of the Paleozoic era, at least 300 million years ago. They are widely distributed across the world except in polar regions, with the greatest diversity found in tropical and subtropical areas. China has a significant advantage in terms of species diversity within the Odonata, with over 1000 species discovered so far. This includes a large number of endemic and endangered species, which are of high conservation value. Dragonflies are common resource insects and are important flagship species and environmental indicators [2]. In the tropics, they feed on a wide range of disease-carrying mosquitoes, providing beneficial biological control and an extremely high utility value. They require high quality aquatic and forest vegetation habitats, and their sensitive characteristics are often used for ecological environmental assessment.

Recording and comparing species from different geographical locations for large-scale analysis is an extremely difficult task. Several databases have been developed to address this challenge. Integrated online databases include the Atlas of Living Australia (https://www.ala.org.au/), iNaturalist (http://www.inaturalist.org/), the IUCN Red List (http://www.iucnredlist.org/), and VertNet [3], all of which have rich geographical, physiological, and biochemical information, and have now become important sources for large-scale analysis [4]. However, there are very few online databases focused on invertebrates (especially insects). To meet the growing demand, more and more insect data are being digitized in online repositories, and the number of online invertebrate databases is gradually increasing, such as FreshWaterBiodiversity (http://data.freshwaterbiodiversity.eu/), Global Biodiversity Information Facility (http://www.gbif.org/), Odonata Central, and the Odonate Phenotypic Database [5]. In addition to global-scale online biodiversity databases, a growing number of regionally focused platforms—such as Butterflies of India (https://www.ifoundbutterflies.org/), Butterflies of Belgium, Odonata of India (https://www.indianodonata.org), the British Dragonfly Society (https://british-dragonflies.org.uk/), the Hong Kong Agriculture, Fisheries and Conservation Department (https://sc.afcd.gov.hk/), Odonata of Bangladesh (http://www.odobd.org), and the Illinois State Museum’s Odonata database have emerged (http://www.museum.state.il.us/). These repositories provide granular spatiotemporal data on extant species, offering critical insights for regional ecological and taxonomic studies.

The Bangladesh Odonata Database (http://www.odobd.org) and the Illinois State Odonata Database (USA) are prominent examples of regional biodiversity repositories. The Bangladesh Odonata Database (http://www.odobd.org) aims to collate and disseminate comprehensive data on the diversity, ecology, and behaviour of dragonflies and damselflies in Bangladesh. Its successful establishment provides a platform for conservationists and the general public to enhance their understanding of Odonata and encourages active participation in the protection of these insects and their habitats. The database integrates morphological descriptions, abundance metrics, flight seasonality, genetic and protein sequences, local and global distribution patterns, conservation statuses, and sex-specific photographic records. Regular updates ensure its continued relevance as a critical resource for documenting Bangladesh’s Odonata diversity and distribution, which is essential for assessing extinction risks and formulating targeted conservation strategies. Furthermore, the database facilitates global Odonata analyses in collaboration with other regional platforms, enabling insights into macroecological patterns and climate change impacts. The Illinois State Odonata Database compiles over 6500 records sourced from institutional and private collections, encompassing ~150 species across 9 families of dragonflies and damselflies. These records predominantly originate from 92 counties within the USA. The database catalogues species-specific data on distribution, ecology, behavioural traits, and genetic variation. The managing team is committed to advancing the conservation of Odonata species across the USA through evidence-based research and public engagement.

China represents the most species-rich nation for Odonata (dragonflies and damselflies) globally, with over 900 documented species. However, the extensive fragmentation of unintegrated data poses significant challenges to large-scale analyses and comprehensive research on Chinese Odonata. To address this critical gap, there is an urgent need to establish an online database consolidating the majority of known Odonata species in China. Such a platform would serve as a centralized, openly accessible resource to facilitate research advancements in evolutionary biology, ecology, taxonomy, and conservation science for this ecologically vital insect group.

Here, we constructed the Odonata of China Database (http://dragonflies.kiz.ac.cn/), a comprehensive repository of taxonomic, ecological, and distributional data for dragonflies and damselflies across China. To date, the database has systematically compiled information on 820 distinct species documented nationwide, integrating critical contributions from peer-reviewed literature [6–33]. The Odonata of China Database integrates comprehensive data on morphological characteristics, body length, habitat preferences, global and regional (China-specific) distributions, flight periods, barcode information (e.g. DNA barcodes), conservation statuses, literature references, and sex-specific photographic records. Notably, the database also incorporates multiomics datasets, including whole genomes, transcriptomes, and mitochondrial genomes of Odonata species. Periodic updates ensure the platform remains current with advancing research. This repository serves as an indispensable resource for evaluating extinction risks and informing evidence-based conservation strategies for China’s Odonata. Furthermore, in conjunction with other regional and global databases, it supports large-scale analyses to investigate macroecological patterns and the ecological impacts of climate change on this taxon. The Odonata of China Database aims to become the most comprehensive and authoritative reference database for the Odonata in China. By establishing standardized protocols and a data integration mechanism, it extensively collects and integrates multidimensional data on Chinese Odonata. The database is being developed to provide key data support for the diversity of Chinese Odonata, extinction risk assessment, and the protection needs of endangered Odonata.

## Materials and methods

### Data collection and curation

To ensure the diversity and comprehensiveness of our dataset, we integrated multisource data collected from an extensive review of scientific literature and specialized monographs. Key references include works by Dr Hao-Miao Zhang, including ‘*Dragonflies and Damselflies of China*’, ‘*A Photographic Guide to Dragonflies of China*’, ‘*New Insect Chronicles—Dragonfly Flight Diary*,’ and ‘*A Field Guide to the Dragonflies of Hainan*’. To ensure data accuracy and reliability, a systematic quality control protocol was implemented: data were initially extracted and processed from dragonfly field guides and online databases using automated scripts and optical character recognition technology, followed by automated cleansing including terminology standardization, logical consistency checks, and deduplication; subsequently, multitiered validation was performed via randomized manual sampling and blind review by domain experts to verify critical information, such as species identification and distribution records; finally, version control and continuous update mechanisms were adopted to maintain data currency and traceability.

These works provide detailed information on dragonfly morphology, ecological habits, distribution locations, and more, forming a solid foundation for our dataset. Our research integrates dual data is further supplemented by systematic field surveys, which augment the breadth of ecological data through standardized protocols. These surveys yield critical insights into taxon-specific habitat preferences, phenological patterns (e.g. seasonal distribution), and microhabitat utilization. Complementing primary field data, we integrate curated observations from open-access biodiversity platforms such as iNaturalist and the IUCN Red List, which provide: (i) georeferenced photographic records; (ii) crowd-sourced occurrence records from researchers and citizen scientists; and (iii) standardized metrics of species conservation status. This multisource approach not only enhances the robustness of our dataset but also enables cross-validated ecological patterns and multiscale analytical frameworks for Odonata research.

Our study compiled a comprehensive dataset encompassing 820 Odonata taxa (species and subspecies) across a hierarchical classification of three suborders, 22 families, and 172 genera: Anisoptera (13 families, 65 genera, and 293 species), Zygoptera (1 family, 1 genus, and 3 species), and Anisozygoptera (8 families, 106 genera, and 524 species) (Table 1). The core of the database consists of species information, which has achieved near-complete coverage of all known dragonfly species in China. It will be continuously updated to incorporate newly described species in the future. Quantitatively, the dataset contains expertly verified image records for 814 species, representing 99.3% coverage of currently recognized Odonata taxa in China. In parallel, it comprises DNA barcode sequences corresponding to 309 distinct species, achieving a taxonomic coverage of 37.68% of known Odonata diversity within the country. This core taxonomic resource is further enriched by an extensive repository of crowd-sourced photographic contributions, which bolster the ecological and morphological breadth of the database.

**Table 1.** The statistical information of the datasets included in Odonata of China database

Suborder	Families	Genus	Species
Anisoptera	13	65	293
Zygoptera	1	1	3
Anisozygoptera	8	106	524
Total	22	172	820

### Implementation and deployment details

For data management, we implemented a tiered storage architecture optimized for both structured and unstructured biological data. Structured species metadata, including taxonomic classifications, morphological measurements, and ecological parameters that is housed in a normalized MySQL relational database (https://www.mysql.com/), designed with ACID-compliant transaction support to ensure data integrity and query efficiency. The schema employs entity-relationship modelling, with tables for families, genera, species, and observational records linked through foreign key constraints, enabling complex JOIN operations for phylogenetic and biogeographic analyses. Unstructured image assets, such as high-resolution specimen photographs and habitat panoramas, are managed via MinIO (https://www.minio.org.cn/) object storage, a cloud-native S3-compatible system selected for its horizontal scalability and metadata tagging capabilities. Each image file is stored as a binary large object with associated metadata (e.g. Latin binomials, geospatial coordinates, and photographer credits) indexed in MySQL through UUID-prefixed storage paths. This hybrid architecture decouples computationally intensive image processing from transactional database operations while maintaining referential integrity via programmatic synchronization.

**Figure 1.**
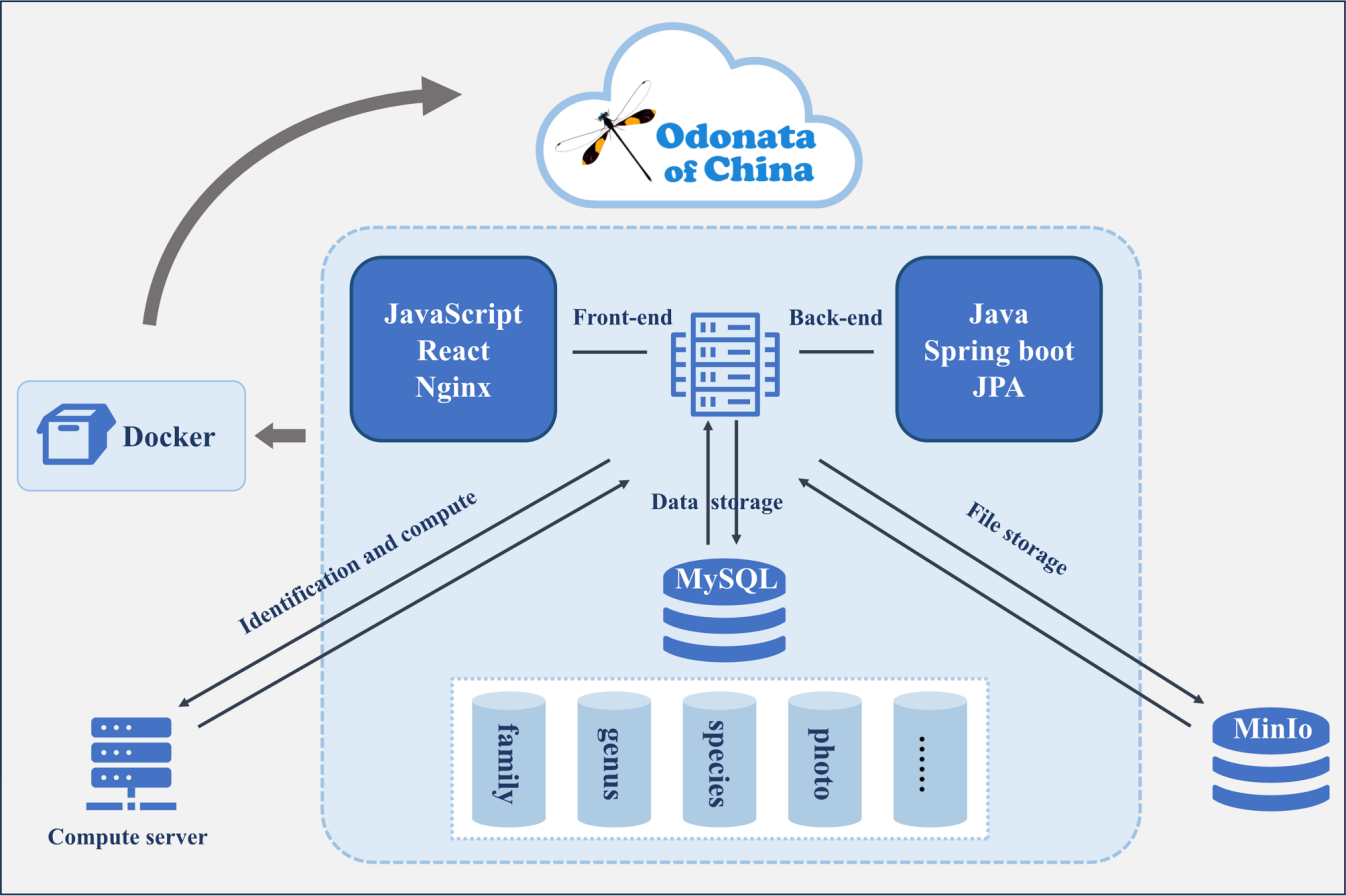
The overall functionality of the database is mainly focused on search, browsing, sequence alignment, image display, and contact information. It adopts a front-end and back-end separation technology, with the front-end and back-end respectively paired with the mainstream development frameworks React and Spring Boot. The database uses MySQL, and uses high-performance object storage MinIO for file storage, with the main file type being images. The entire database is encapsulated using Docker.

The Odonata of China Database’s frontend leverages React (https://react.dev/), a component-based JavaScript framework that modularizes user interface development, enabling dynamic visualization of ecological and morphological data through reusable UI components. The backend, built with Java and Spring Boot (https://spring.io/projects/spring-boot), provides RESTful APIs for data access and business logic, while Docker (https://www.docker.com/) containerization ensures consistent deployment across Linux environments, resolving dependency conflicts and enabling horizontal scaling (Fig. 1). This architecture balances computational efficiency, maintainability, and interoperability, aligning with FAIR principles for biodiversity data stewardship.

The Odonata of China Database supports most of the widely used web browsers including Google Chrome, Mozilla Firefox, and Microsoft edge.

## Results

### Overview of Odonata of China database

**Figure 2.**
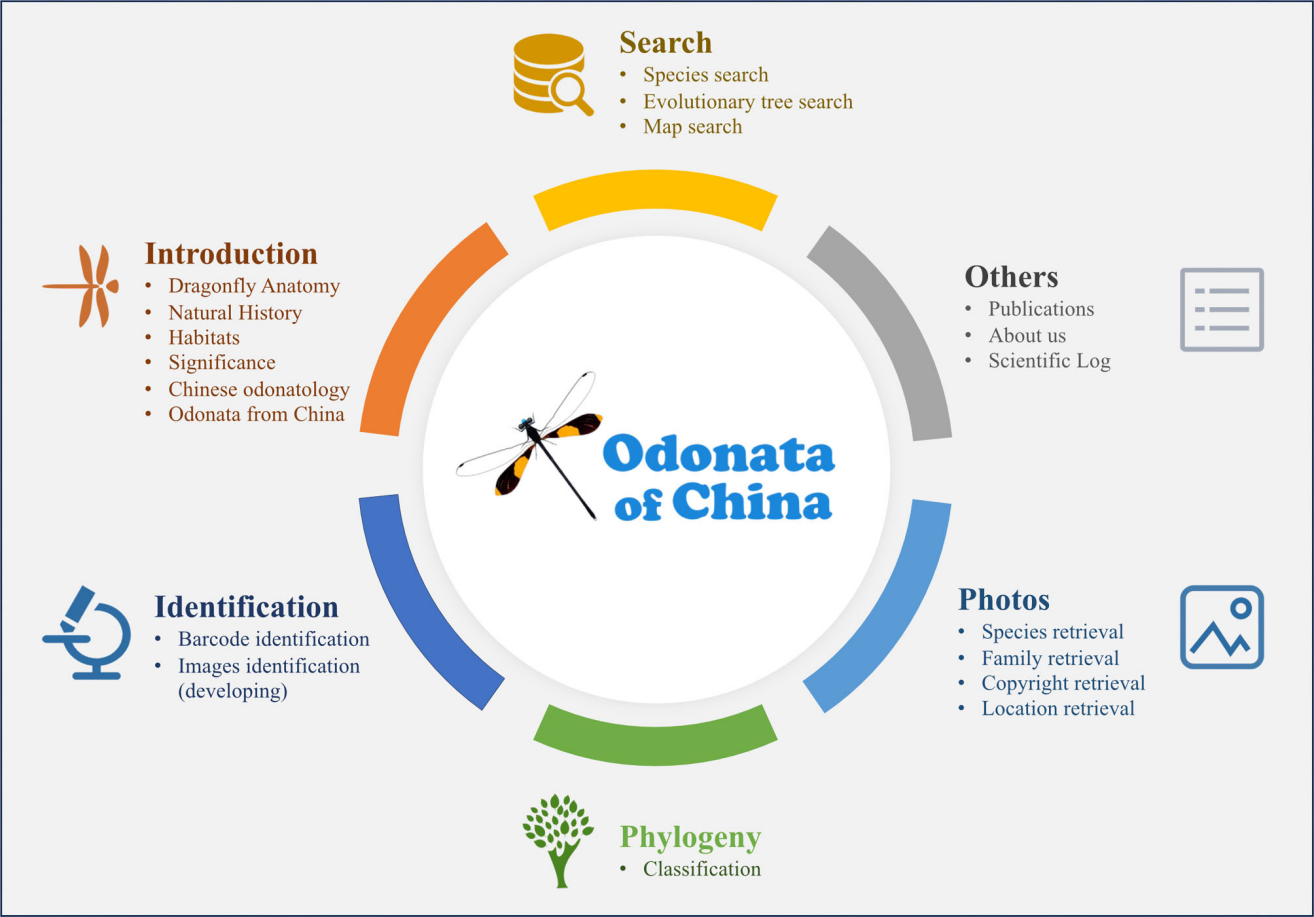
An overview of Odonata of China database. The main data contents and statistics including search, identification, photos, and phylogeny.

The Odonata of China database consists of six modules as shown in Fig. 2: search, introduction, identification, phylogeny, photos, and others. The search module focuses on species search, evolutionary tree search, and map search. The introduction module mainly provides information about dragonfly habitats and Odonata anatomy. The identification module is primarily concerned with DNA barcode-based identification and morphological image-based identification. The phylogeny module presents the classification of Odonata in China. The photos module primarily displays dragonfly images, allowing users to search by species name, family name, and location of the photos. The others module mainly showcases contact information, expedition logs, publications, and similar content.

### Web content and usage

The home page first includes a navigation bar that can be used to jump to modules such as introduction, phylogeny, and species. It also adds a quick search bar that can be used to search for dragonfly species by their Latin name and jump to the corresponding species information page. The homepage also features six popular dragonfly species from around the world with pictures and text. Clicking on a species will take you to its detailed page. The homepage also has information on dragonfly records in China and a distribution map that shows the distribution of dragonflies in different provinces in China. Clicking on a province will show the specific species distributed in that area. Within home page, six globally renowned odonate groups are showcase: Genus Anax Leach, 1815; Genus Rhyothemis Hagen, 1867; Family Chlorocyphidae; Family Chlorogomphidae; Helicopter Damselflies (Pseudostigmatidae); and Metalwing Demoiselles (Calopterygidae: *Neurobasis* spp.). These taxa are highlighted for their distinctive physiological adaptations—such as hypertrophied wing venation in Chlorocyphidae, aerial predation strategies in Anax, and iridescent cuticular nanostructures in Metalwing Demoiselles, which have garnered significant scientific and ecological attention for their evolutionary novelty and ecological specialization.

### Search

**Figure 3.**
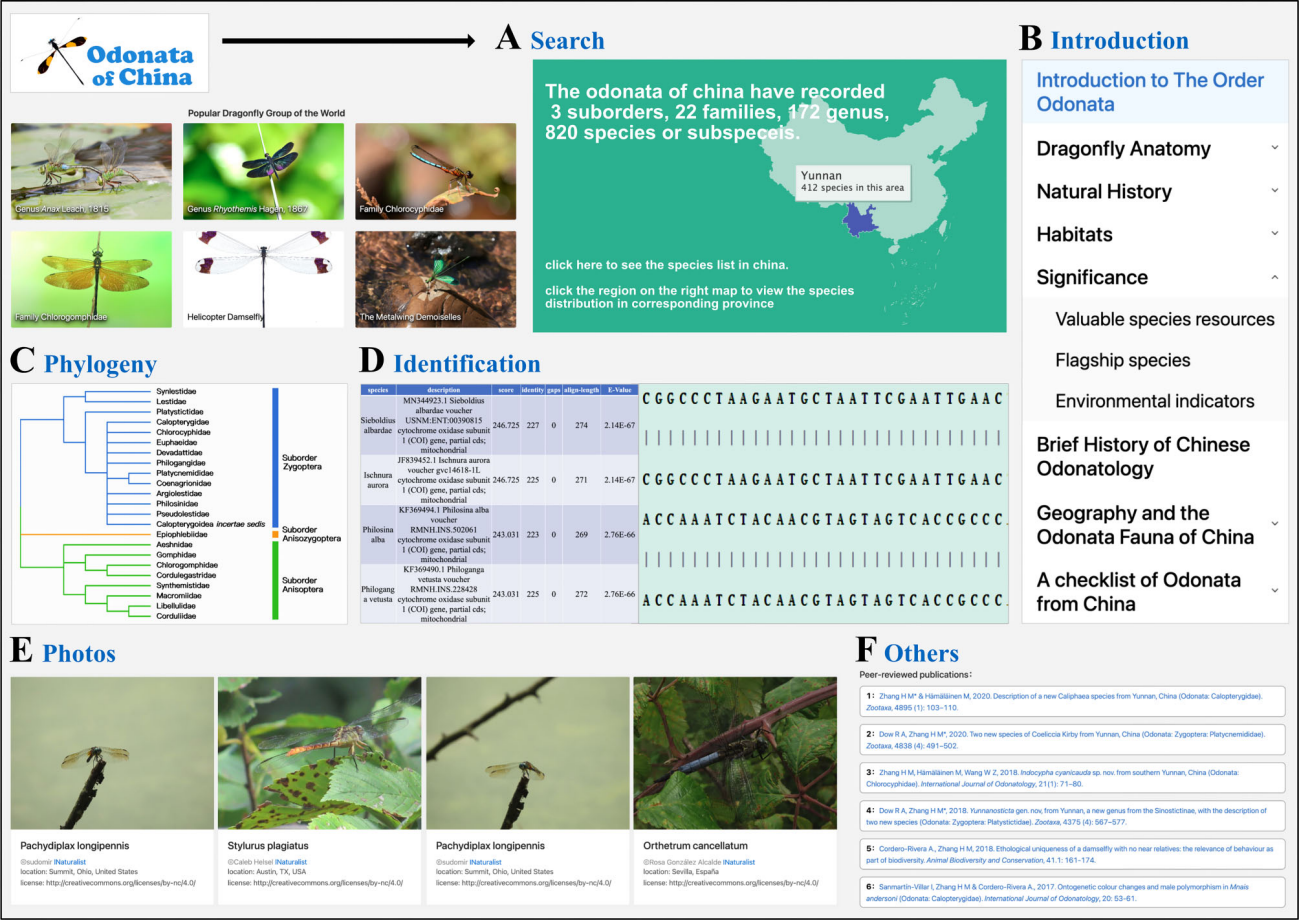
Illustration of browse and search interfaces in Odonata of China database. (A) The search web interface of Odonata of China database. (B) The introduction web interface. (C) The phylogeny web interface. (D) The identification web interface. (E) The photo display page. (F) Display the page of the published paper.

The search function allows users to search by the Latin name of a species, with the system providing autocomplete suggestions based on the user’s input. Additionally, users can search the phylogenetic tree by family name, which then links to the genus name, and from the selected genus, it links to the species name, ultimately helping users find the desired species. The map search is primarily based on the distribution of Odonata across regions. Users can click on different provinces in China to filter results, and the search results will display the species of Odonata found in the selected province (Fig. 3A).

Users with prior knowledge of scientific names can directly query the China Odonata Database to retrieve taxon-specific profiles. For instance, entering the Latin binomial *Neurothemis fulvia* into the navigation bar’s search interface returns a comprehensive entry detailing morphological traits of species, ecological preferences, genomic resources, and georeferenced distribution records within China. This functionality employs fuzzy-matching algorithms to accommodate minor orthographic variations (e.g. misspelled genus prefixes), ensuring robust accessibility for both taxonomic experts and citizen scientists.

When users want to search through the evolutionary relationships of a certain species, they can search according to the evolutionary tree in the database. Take ‘*Neurothemis fulvi*’ as an example. The order—level classification of *Neurothemis fulvia* is ‘Anisoptera’, the family—level classification is ‘Libellulidae’, and the genus—level classification is ‘*Neurothemis*’. First, the user finds ‘Anisoptera’ on the evolutionary tree and clicks on it to enter the next page. Second, find ‘Libellulidae’ on the new page and click on it to enter the next page. Then, find ‘*Neurothemis*’ on the new page and click on it to enter the next page. Finally, find ‘*Neurothemis fulvia*’ on the new page and click on it to enter the page introducing the species.

Users wish to search for a species based on its geographical distribution, they can utilize the map on the database. Taking ‘*Neurothemis fulvia*’ as an example, this species is primarily distributed in the following Chinese provinces: Yunnan, Fujian, Guangdong, Guangxi, Hainan, Hong Kong, and Taiwan. We demonstrate the search process using its distribution in Yunnan. First, users locate ‘Yunnan’ on the map and click to access the next page. Second, they find ‘Libellulidae’ on the new page and click to proceed. Then, they identify ‘*Neurothemis*’ on the subsequent page and click to advance. Finally, they locate ‘*Neurothemis fulvia*’ on the resulting page and click to enter the species' detailed introduction page.

### Introduction

The introduction module of the Odonata of China Database is structured into eight thematically organized sections to systematically disseminate foundational knowledge on Odonata biology: order overview, morphological anatomy, natural history, habitat ecology, chinese biogeography, conservation status, behavioural adaptations, and taxonomic classification. Each section employs a multimodal approach, integrating peer-reviewed textual summaries with high-resolution anatomical schematics, habitat photographs, and GIS-mapped species distribution overlays to elucidate key topics such as provincial endemism patterns (e.g. *Epiophlebia sinensis* in Sichuan), larval microhabitat preferences, and evolutionary adaptations. Interactive elements—including annotated 3D models of wing venation and clickable hotspot maps of biodiversity-rich regions like Hainan Island—enhance data accessibility, while citations to authoritative sources ensure alignment with current scientific consensus. This pedagogical framework bridges public education and academic research, reinforcing the database’s role as a comprehensive resource for Odonata biodiversity in China (Fig. 3B).

### Phylogeny and species

The ‘Phylogeny’ section displays the taxonomic information of the three suborders of Odonata in China, including 23 families. Selecting different families will display an introduction to that family and all the genera under that family. Selecting different genera will display basic information about that genus and all the species under that genus. Clicking on a species will display detailed information about that species, including identification information, measurement information, Flight Season, Habitat, distribution information, and more. As shown in Fig. 3(C), According to the latest research results of Dijkstra [34, 35] and Carle [36], the Odonata of China are divided into 3 suborders and 23 families. Blue represents the suborder Zygoptera, which has a total of 14 families. Orange represents the suborder Anisozygoptera, which has a total of 1 family. Green represents the suborder Anisoptera, which has a total of 8 families.

### Identification

The ‘Identification’ section mainly includes the function of sequence alignment. Users can input a sequence for nucleotide alignment. The alignment results are displayed in a table format, and the information in the table includes species Latin name, description, score, etc. At the same time, the alignment results of different species with the input sequence are displayed at the bottom of the table. Clicking on the Latin name of a species in the table will jump to the detailed information of the species, and clicking on the description content will display the detailed sequence alignment of the corresponding species. As shown in Fig. 3(D), the data chart shows the result information after comparison, including species, description, score, identity, gaps, align-length, and E-Value. Clicking on species and description can jump to the corresponding detailed information.

### Photos and others

‘Photos’ integrates dragonfly information from iNaturalist, presenting dragonflies in a gallery format with images and accompanying text (Fig. 3E). It also provides a search function, where users can retrieve dragonfly photos based on species Latin name, family name, image uploader, and location. ‘Publications’ primarily lists information about published papers and books (Fig. 3F). Users can contact us through the contact information provided in the ‘About Us’ section.

## Discussion and future development

The Odonata of China database provides a platform for convenient browse and utilize the massive data produced by integrating phenotypic, ecological, and multiomics. Our team has collected a total of 820 species (including subspecies) from 3 suborders, 22 families, and 172 genera of dragonflies in China, accounting for 83.42% of the total known dragonfly species in China. Among them, the suborder Anisoptera includes 13 families, 65 genera, and 293 species; the suborder Zygoptera includes 1 family, 1 genus, and 3 species; the suborder Epiprocta includes 8 families, 106 genera, and 524 species. Key data information on dragonfly species, such as morphological characteristics, length, habitat, distribution in the world and China, flight period, barcode information, survival status and protection level, references, and representative photos, etc. Furthermore, to enhance the replicability and cross-regional applicability of our digitization framework, we have formalized a comprehensive standardized cataloguing protocol for Odonata of China database, detailing metadata annotation, this specification document is provided in ​Appendix 1​ to serve as a scalable reference for global biodiversity digitization initiatives. The Odonata of China database has multiple search methods, including direct search, phylogenetic tree search, and map search. In addition, the database has very exquisite and rich graphics and texts, showing the original colour ecology of each dragonfly, important classification features such as wing veins and external genitalia structure, and truly presents the brilliant and colourful, varied and diverse morphological structure of dragonflies, providing users with an interesting and friendly information resource library. The Odonata of China database has been completed and is available for free use. In the follow-up work, the Odonata of China database team will further update it according to related research results and try to add more functions.

The storage and access of large-scale scientific research datasets can be achieved through database. These databases can carry out large-scale ecological and evolutionary analyses, which are an important part of understanding macroevolutionary processes. To meet these needs, we have developed a comprehensive database for the Odonata of China. We have collected phenotypic (body size, wing size, body colour, and wing colour), taxonomic (spectroscopy, descriptive, gene and protein sequences, identification keys), biogeographical (regional and global distribution), taxonomic data, and genomic data of the Odonata of China.

Comparative analysis is an important part of understanding the evolution of phenotypic traits and requires a large amount of phenotypic data. Researchers have studied the body size and wing size of Odonata to understand the relationship between size and geographical latitude [37, 38]. Our database provides body size and wing size data for 820 species, which facilitates future studies on the micro and macro evolutionary patterns of body size and the determination of extinction risk [39]. With the continuous development of modern geographic information systems (GIS), the accumulation of a large amount of data can help us understand the habitat requirements of species and can further help us understand the impact of climate change on species habitats, which is particularly important for Odonata with short life cycles and strict habitat requirements.

Digital photos are a powerful medium for studying animals, ecology, and evolutionary significance [40]. Recent research has shown the importance of photography websites for studying animals, ecology, and evolution [41, 42]. In addition, many Odonata exhibit colour polymorphism, which reduces sexual conflict and improves population fitness [43, 44].

The photos of different colour forms we have accumulated can be used for research on colour polymorphism. Photos of Odonata at different developmental stages help to study the genetic colour of Odonata, which is also related to the sexual maturity of Odonata [45, 46]. It is noteworthy that our team has recently made significant progress in applying computer vision technology to advance the taxonomic study of dragonflies. In a study published in Applied Sciences, we successfully migrated the ArcFace and ResNet models from face recognition to an insect classification task, markedly improving the automatic identification accuracy of dragonfly images [47]. This approach provides critical technical support for the image retrieval and species identification functions of the present database, further enhancing the mineability and application value of the data.

This database will become a valuable resource for understanding the ecology, extinction risk, and conservation importance of Odonata in China. In addition, the database can be used for comparative analysis to understand the patterns and processes of macroecology and biogeography. The photos of male and female insects at different developmental stages, as well as the colour forms of different species that we have saved, will be a valuable resource for studying colour functions. We will continue to update our database, monitor the impact of climate change on Odonata, and the extinction risk of Odonata. With the rapid development of big data research in science, traditional Odonata species names and species cataloguing can no longer solve the big data based on Odonata biodiversity research. The construction of the Odonata of China database integrates and generates scientific data closely related to human needs and the laws of discipline development from the three levels of Odonata’s ecology, species, and genes. It connects scattered and fragmented regions to ensure the connectivity and integrity of regional ecosystems.

Multiomics data represent a pivotal frontier in odonate research, and our team has currently integrated comprehensive multiomics datasets encompassing whole genomes, transcriptomes, and mitochondrial genomes from representative species across 20 families, including Devadattida, Calopterygida, Chlorocyphidae, Euphaeida, Philogangida, Philosinida, Argiolestida, Pseudolestida, Lestida, Synlestida, Platycnemididae, Coenagrionida, Platystictida, Aeshnida, Gomphida, Chlorogomphid, Cordulegastrida, Corduliida, Macromiida, Synthemistida, and Libellulida. The next phase involves incorporating these omics resources into our centralized database to enable comparative genomic analyses, phylogenomic reconstructions, and conservation genomic applications for Odonata research.

The Odonata of China database will provide support services for scientists in the field of Chinese Odonata, continuously promote the exchange of interlinked data, gradually promote the integration of paper-related data, use the international dissemination of papers to enhance the international influence of Chinese Odonata scientists. In addition, the Odonata of China database will also actively participate in the related work of the International Odonata Society, enhance the influence of the database internationally, and demonstrate a sense of international responsibility.

## Supplementary Material

baaf077_Supplemental_File

## Data Availability

All data used in the analysis can be obtained at https://dragonflies.kiz.ac.cn/.
