# Supplementary material for: The Odonata of China: a data-driven, open-access resource for biodiversity research and conservation
Source: Database (Oxford). 2025 Dec 23;2025:baaf077. doi: 10.1093/database/baaf077 (PMC12724073; doi:10.1093/database/baaf077)
Supplement: baaf077_Supplemental_File [file baaf077_supplemental_file.doc]

**China dragonfly cataloging standards and specifications**

Contents

[Dragonfly Standard Cataloging 1](#__RefHeading___Toc209975403)

[1 Species ID 1](#__RefHeading___Toc209975404)

[2 Scientific Name 1](#__RefHeading___Toc209975405)

[3 Species 2](#__RefHeading___Toc209975406)

[4 Author 2](#__RefHeading___Toc209975407)

[5 Year 2](#__RefHeading___Toc209975408)

[6 Suborder 2](#__RefHeading___Toc209975409)

[7 Family 2](#__RefHeading___Toc209975410)

[8 Genus 3](#__RefHeading___Toc209975411)

[9 Morphological Characters 3](#__RefHeading___Toc209975412)

[10 Measurements 3](#__RefHeading___Toc209975413)

[11 Flight Season 4](#__RefHeading___Toc209975414)

[12 Habitat 4](#__RefHeading___Toc209975415)

[13 Distribution 4](#__RefHeading___Toc209975416)

[14 Domestic Distribution 4](#__RefHeading___Toc209975417)

[15 Global Distribution 4](#__RefHeading___Toc209975418)

[16 DNA Barcode 5](#__RefHeading___Toc209975419)

[17 Conservation Status 6](#__RefHeading___Toc209975420)

[18 References 6](#__RefHeading___Toc209975421)

# Dragonfly Standard Cataloging

The Dragonfly Standard Cataloging Framework was developed with reference to the Darwin Core standard, establishing a structured dataset for the Odonata of China database that comprises 18 standardized fields. It systematically organizes: Species ID, Scientific Name, Species, Author, Year, Suborder, Family, Genus, Morphological Characters, Measurements, Flight Season, Habitat, Distribution, Domestic Distribution, Global Distribution, DNA Barcode, Conservation Status and References.

## 1 Species ID

Mandatory.

Character type.

Species ID.

Unique identifier for each species record within the database system.

To minimize duplication, adopt a consistent naming convention, e.g., institutional abbreviation + author initials + sequential number.

Example: KIZZHM001

## 2 Scientific Name

Mandatory.

Character type.

Scientific name as displayed on the database webpage.

Example: Archineura hetaerinoides (Fraser, 1933)

## Species

Mandatory.

Character type.

Binomial nomenclature of the species.

Example: *Archineura hetaerinoides*

## Author

Mandatory.

Character type.

Original discoverer of species description.

Example: Fraser

## Year

Mandatory.

Datae type.

Original discovery year of species description.

Example: 1933

## Suborder

Mandatory.

Character type.

Taxonomic suborder classification.

Example: *Zygoptera*

## Family

Mandatory.

Character type.

Taxonomic family name in Latin.

Example: *Calopterygidae*

## Genus

Mandatory.

​Character type.

Taxonomic genus name in Latin.

Example: *Archineura*

## Morphological Characters

​Mandatory.

Character type.

Descriptive text of diagnostic morphological features.

Example: Male face dark green, labrum with yellow spots. Thorax and abdomen metallic dark green, wings hya-line with cream white patches at base, side of synthorax with slight pruinescence in old males. Female wings slightly tinted with brown. Males from the type locality possess cream white patches only on hind wings, identical to the population in the west of Yunnan. Males from Guangxi and the east of Yunnan possess cream white patches on both wings, size slightly larger.

## Measurements

​Mandatory.

​Character type.

Quantitative morphological measurements.

Example: Total length 80-87 mm, abdomen 64-69 mm, hind wing 49-54 mm.

## Flight Season

Mandatory.

Character type.

Active flight period of the species.

Example: April to July.

## Habitat

Mandatory.

Character type.

Ecological description of preferred habitats.

Example: Open and rocky streams in forested area below 1200 m elevation.

## Distribution

Mandatory.

Character type.

Geographic distribution range.

Example: Guangxi, Yunnan; Burma, Laos, Vietnam.

## Domestic Distribution

Mandatory.

Distribution within China.

​Character type.

Example: Guangxi and Yunnan.

## Global Distribution

Mandatory.

​Character type.

Worldwide distribution range.

Example: Myanmar、Laos、Vietnam、China

## DNA Barcode

Mandatory.

Character type.

DNA barcode sequence.

Example: >MN344958.1 Archineura incarnata voucher USNM:ENT:00390998 cytochrome oxidase subunit 1 (COI) gene, partial cds; mitochondrial

TACTTTATATCTCTTATTCGGAGCATGGGCTGGAATAGTGGGAACTGCTTTAAGAATAATAATCCGAGTT

GAACTCGGACAGCCGGGGTCACTCATTGGTGACGACCAAATCTATAACGTAATAGTGACTGCGCACGCAT

TTGTTATAATTTTCTTTATAGTTATACCAATTATAATTGGTGTGTTCGGTAACTGATTGGTGCCCCTGAT

ACTTGGGGCGCCCGATATAGCTTTCCCCCGACTAAATAACATAAGATTCTGATTACTCCCTCCCGCATTA

ACATTACTACTAACAAGAAGTTTAGTAGAAAGAGGGGCGGGAACAGGGTGGACAGTCTACCCGCCCTTAG

CAAGAAACATCGCGCACACGGGCGGGTCAGTAGACCTGACCATTTTCTCACTACACCTAGCGGGTATATC

ATCTATATTAGGAGCTATTAACTTCATTACCACAACGATTAATATGAAGTCCCCAGGAATAAAAATGGAT

CAGATACCCCTATTCGTCTGAGCCATATTNATTACTGCAATTCTACTACTTCTATCACCCCCCGTATTGG

CAGGAGCCATTACAATACTACTAACTGACCGAAACTTAAACACCTCCTTTTTTGATCCTGCTGGGGGTGG

TGATCCGATTTTATATCAACACCTATTT

## Conservation Status

​Mandatory.

Character type.

IUCN Red List category.

Example 1: Least Concern

Example 2: Near Threatened

## References

Mandatory.

Character type.

Key literature citations.

Example: 1. Zhang, H-M. 2019. Dragonflies and Damselflies of China. Chongqing: Chongqing University Press, China.
